# Mice Chronically Fed High-Fat Diet Have Increased Mortality and Disturbed Immune Response in Sepsis

**DOI:** 10.1371/journal.pone.0007605

**Published:** 2009-10-28

**Authors:** Louise Strandberg, Margareta Verdrengh, Maria Enge, Niklas Andersson, Sylvie Amu, Karin Önnheim, Anna Benrick, Mikael Brisslert, Johan Bylund, Maria Bokarewa, Staffan Nilsson, John-Olov Jansson

**Affiliations:** 1 Institute of Neuroscience and Physiology/Endocrinology, The Sahlgrenska Academy at the University of Gothenburg, Gothenburg, Sweden; 2 Department of Rheumatology and Inflammation Research, University of Gothenburg and Sahlgrenska University Hospital, Gothenburg, Sweden; 3 Department of Mathematical Statistics, Chalmers University of Technology, Gothenburg, Sweden; University of Liverpool, United Kingdom

## Abstract

**Background:**

Sepsis is a potentially deadly disease that often is caused by gram-positive bacteria, in particular *Staphylococcus aureus* (*S. aureus*). As there are few effective therapies for sepsis, increased basic knowledge about factors predisposing is needed.

**Methodology/Principal Findings:**

The purpose of this study was to study the effect of Western diet on mortality induced by intravenous *S. aureus* inoculation and the immune functions before and after bacterial inoculation. Here we show that C57Bl/6 mice on high-fat diet (HFD) for 8 weeks, like genetically obese Ob/Ob mice on low-fat diet (LFD), have increased mortality during *S. aureus-*induced sepsis compared with LFD-fed C57Bl/6 controls. Bacterial load in the kidneys 5–7 days after inoculation was increased 10-fold in HFD-fed compared with LFD-fed mice. At that time, HFD-fed mice had increased serum levels and fat mRNA expression of the immune suppressing cytokines interleukin-1 receptor antagonist (IL-1Ra) and IL-10 compared with LFD-fed mice. In addition, HFD-fed mice had increased serum levels of the pro-inflammatory IL-1β. Also, HFD-fed mice with and without infection had increased levels of macrophages in fat. The proportion and function of phagocytosing granulocytes, and the production of reactive oxygen species (ROS) by peritoneal lavage cells were decreased in HFD-fed compared with LFD-fed mice.

**Conclusions:**

Our findings imply that chronic HFD disturb several innate immune functions in mice, and impairs the ability to clear *S. aureus* and survive sepsis.

## Introduction

Sepsis is often a deadly disease with increasing incidence worldwide [1]. Gram-positive bacteria, in particular *Staphylococcus aureus* (*S. aureus*), are a predominant and increasing cause of sepsis [1], [2]. Sepsis consists of a hyperinflammatory state during the first few days followed by a prolonged hypoimmune state during which death often occurs [3], [4]. At present, there are few treatments for sepsis, besides antibiotics [3], [5], and antibiotic resistance is increasing globally [6]. In addition, few treatments for sepsis developed in experimental animals have resulted in clinical use [4], [5]. Therefore, increased basic knowledge about factors predisposing to this deadly disease is urgently needed.

Like sepsis, obesity is increasing epidemically and this is believed to be due to the consumption of energy dense food, such as fat rich diets, combined with polygenetically determined susceptibility [7], [8]. There may be interactions between the immune system and body fat metabolism. The adipose tissue of obese and insulin resistant individuals can produce and release cytokines, first shown by Hotamisligil & Spiegelman for tumor necrosis factor (TNF)-α [9]. More recently, it has been suggested that macrophages accumulating in fat [10], [11] contribute to obesity-induced cytokine release and low-grade inflammation, which in turn could cause insulin resistance and atherosclerosis in obese subjects [10]–[12]. Considerably less is known about how this condition may influence the main task of the immune system, to combat infections. Clinical findings indicate that obesity is associated with increased susceptibility to infections [13]. However, this association could be due to multiple factors, e.g. longer surgery and hospitalization time of obese patients, with increased risk for nosocomial infections [13]. Alternatively, obesity could be secondary to immune defects. Previous studies have shown that innate immune dysfunction, associated with absence of interleukin-6 (IL-6), granulocyte-macrophage colony-stimulating factor, IL-1RI, and IL-18, leads to obesity [14]–[19]. Conversely, ingestion of energy dense food and obesity may suppress the immune response. In clinical materials it is very difficult to clarify the possible causality as well as cellular and molecular links between obesity and potential defective immune function.

In the present study we used an animal model to determine whether ingestion of fat with resulting obesity can affect the capacity of the immune system to resist bacterial sepsis. We studied chronic high-fat diet (HFD) fed mice, which have been shown to reflect common forms of clinical obesity and obesity related disease that depend on multiple genetic factors as well as diet [20], [21]. The bacterial challenge model used in this study was injection of *S. aureus*, a common cause of sepsis in humans.

## Methods

### Ethics statement

The experiments were performed with the approval of the Ethical Committee of University of Gothenburg.

#### Experimental protocol

Male C57BL/6 mice 5–7 weeks old were obtained from Harlan Netherlands B.V. (Horst, The Netherlands). Male Ob/Ob mice and C57BL/6 control mice were obtained from Harlan UK Limited (Bicester, England). The mice were housed in the animal facility of the Department of Rheumatology and Inflammation Research or at the Laboratory for Experimental Biomedicine, University of Gothenburg, Sweden, under standard conditions of light and temperature. Food and water were provided *ad libitum*. Starting at 6–8 weeks of age C57BL/6 mice was fed either HFD or LFD for 8 weeks, while Ob/Ob mice were fed LFD. At 14–16 weeks of age the mice were either terminated or infected with *S. aureus*. The experiments were terminated 1 or 5–7 days after inoculation for mechanistic studies and 17 days after bacterial inoculation for observational studies on mortality. Results from different studies were pooled if similar.

The mice received four different diets. LFD R36 (3 kcal/g, 4 g% fat, 18.5 g% protein, 55.7 g% carbohydrate; Lactamin AB, Stockholm, Sweden) was given in experiments with HFD D12309 (5.6 kcal/g, 35.9 g% fat, 23 g% protein, 35.5 g% carbohydrate; Research Diets, New Brunswick, NJ). LFD D12450B (3.85 kcal/g, 4.3 g% fat, 19.2 g% protein, 67.3 g% carbohydrate; Research Diets) was given in experiments with HFD D12492 (5.24 kcal/g, 34.9 g% fat, 26.2 g% protein, 26.2 g% carbohydrate; Research Diets). The two latter diets are matched to have similar content except for fat concentration. There were no obvious differences between the experimental results obtained with these two pairs of diets. In order to study short- versus long-term effects of HFD we studied 4 groups of mice. Mice were fed LFD (LFD→LFD) or HFD (HFD→HFD) before and after bacterial inoculation, we also studied mice on LFD which were switched to HFD on the day of inoculation (LFD→HFD) as well as mice on HFD that were switched to LFD on the day of inoculation (HFD→LFD).

#### *S. aureus* inoculation

Mice were inoculated by an intravenously injection, in the tail vein, with 0.2 ml of *S. aureus* LS-1 solution containing 5×10^7^ colony forming units (CFU), as previously described [22]. After 1 or 5–7 days after inoculation the kidneys were aseptically removed and homogenized in phosphate buffered saline (PBS). Blood was also obtained 1 day following inoculation. Appropriate dilutions were made and 0.1 ml of tissue suspensions or blood was plated on agar plates containing 5% horse blood. After 24 hour incubation at 37°C the staphylococcal colonies were counted.

#### Analysis of body composition

Fat and lean body mass were analysed three days before inoculation by dual-energy X-ray absorptiometry (PIXImus2, Lunar GE Medical systems, Madison, WI). The mice were under inhalation anesthesia with Isoflurane (Forene, Abbot Scandinavia, Solna, Sweden) during the measurement.

#### Serum cytokine measurements

IL-1Ra was measured using enzyme-linked immunosorbent assay (R&D, Abingdon, UK) according to the manufacturer's protocol. IL-10, IL-6, TNF-α, and IL-1β were measured with Bio-Plex mouse cytokine assays (Bio-Rad Laboratories AB, Sundbyberg, Sweden), as previously described [23].

#### mRNA expression

Total RNA from snap frozen liver, spleen and gonadal adipose tissue was extracted using RNeasy Lipid Tissue Mini Kit and RNase-Free DNase Set (Qiagen, Valencia, CA). cDNA synthesis was performed in duplicates using iScript cDNA synthesis kit (Bio-Rad, Hercules, CA). Quantification of mRNA expression was performed using real time reverse transcriptase (RT)-PCR. SYBR Green detection (*Power* SYBR® Green PCR Master Mix, Applied Biosystems, Warrington, UK), was used for *Il1rn*, *Il1b*, *Il10*, and *Tnf*. Melting curves were run to verify PCR products. Probe detection (TaqMan® Gene Expression Master Mix, Applied Biosystems) was used for *Il6* and *Emr1*. The pooled cDNA was run in triplicates along with the following controls: no RT control, negative RT control, and non-template control. The amount of cDNA in each well was equivalent to 50 ng of mRNA and *Eef2* was used as the reference gene. Sequences for primers and assay numbers are shown in Table S1. Gene expression values were calculated based on the ΔΔ*C*_t_ method [24] where the uninfected LFD-fed group was designated the calibrator. Briefly, Δ*C*_t_ represents the threshold cycle (*C*_t_) of the target minus that of the reference gene and ΔΔ*C*_t_ represents the Δ*C*_t_ of each target minus that of the calibrator. Relative quantities were determined using the equation; relative quantity = 2^−^^ΔΔ*C*t^. For the calibrator sample, the equation is relative quantity  = 2^−0^, which is 1; therefore, every other sample is expressed relative to this.

#### Flow cytometry

Peripheral blood was collected in heparinized tubes and total leukocyte counts were determined in a hemacytometer (Toa Medical Electronics, Kobe, Japan). Blood smears were prepared and stained by the May-Grunewald-Giemsa method for differential counts. Spleens were collected and stored in PBS until preparation.

To obtain a spleen single cell suspension for flow cytometry analysis, the spleen was homogenized and run through a nylon filter to remove debris. For removing of erythrocytes blood and cell suspension of spleen were resuspended in NH_4_Cl (0.83%, pH 7.29) and kept on ice for 7 min, washed twice in cold PBS, and finally resuspended in fluorescent-activated cell sorting (FACS)-buffer (containing 1% FCS, 0.1% NaN_3_, 1% EDTA and PBS). Thereafter, 10^5^–10^6^ cells were placed in 96-well plates and pelleted (3 min, 300 *g*, 4°C). Antibodies were diluted in FACS-buffer to optimal concentrations. The monoclonal antibodies used were directly conjugated with allophycocyanin, fluorescein isothiocyanate (FITC), pacific blue, peridinin-chlorophyll-protein or biotin. Antibodies conjugated with biotin were further exposed to streptavidin-conjugated allophycocyanin. Antibodies used included clone 6F12 directed against Emr4 (also called FIRE), and its detection antibody RG7/1.30. Antibodies were also directed against CD19 (1D3), Emr1 (BM8), CD4 (RM4-5), and CD8 (53–6.7). An isotype control for the biotinylated antibody was run at the same time (i.e. rat IgG_2b_, κ,A95-1). In addition, fluorochrome minus one (FMO) settings were also performed and used as background levels for each staining when staining samples with multiple antibodies and fluorochromes [25]. Using FMO settings, the samples are stained with the regular antibodies used in the test covering all flourochromes except one. Analyzing this “empty” channel will give a correct background (i.e. leakage of the other fluorochromes in this channel). This background will then be the base level when analyzing the sample stained with this fluorochrome. As can be seen from the included example of a flow cytometry plot gated with FMO, there is no substantial over- or under-estimation using this method (Fig. S1). This is in line with previous conclusions that FMO settings give lower risk for incorrect gating compared to when isotypes are used [25]. Antibodies were purchased from either BD Bioscience (Erembodegem, Belgium) or E-Bioscience (San Diego, CA).

To avoid unspecific binding via Fc-receptor interactions, cells were incubated with Fc block (2,4G2, BD Bioscience) for 10 min at room temperature. Cells were then incubated with antibodies for a minimum of 20 min at 4°C in the dark followed by two washing steps with cold FACS-buffer. Biotin-conjugated antibodies were incubated in one additional step with streptavidin (allophycocyanin) for minimum of 20 min at 4°C in the dark. After further washing steps cells were resuspended in FACS-buffer before analyses. 2×10^4^−1×10^5^ cells were collected using a FACSCantoII (BD Bioscience). Instrument setting was performed using CompBeads from BD Bioscience and data analyses were performed using the FlowJo software, (Tree Star Inc, Ashland, OR). The background has been subtracted from all presented data.

#### Phagocytic activity analysis

Number and activity of phagocytic cells from whole blood were determined using a commercially available kit (Phagotest®, Orpegen Pharma, Heidelberg, Germany) according to the manufacturer's instructions, except for the use of FITC-labeled *S. aureus*, instead of *Escherichia coli*. In brief, heparinized blood was incubated with FITC-labeled bacteria for 10 min at 37°C, before cooling on ice and addition of quenching solution. Thereafter, the samples are washed, lysed, fixed, washed and stained for DNA, before flow cytometric analysis. The quenching solution of the Phagotest assay allows the discrimination between attachment and internalisation of bacteria by quenching the FITC fluorescence of surface bound bacteria and still leaving the fluorescence of internalized bacteria unaltered.

#### Reactive oxygen species (ROS) production from peritoneal lavage cells

Cells from peritoneal cavity were collected after an injection of 3 ml sterile PBS into the peritoneum followed by a massage of abdomen and collection of the fluid. The cells were then used for measurement of ROS production. An isoluminol-enhanced chemiluminescence (ECL) system was used with a Mithras LB940 (Berthold technologies, Bad Wildbad, Germany) plate reader and disposable 96-well plates containing 200-µl reaction mixtures. Each well contained 2×10^5^ cells, horse-radish peroxidase (4 U ml^−1^) and isoluminol (20 µM) in Krebs-Ringer phosphate buffer (pH 7.3) containing glucose (10 mM), Ca^2+^ (1 mM), and Mg^2+^ (1.5 mM; KRG). The cells were transferred to 37°C, stimulated with phorbol myristate acetate (PMA; 50 nM) or left unstimulated. Light emission was recorded continuously and data are expressed as relative light units; details on the ECL system can be found in [26].

#### ROS production from bone marrow neutrophils

Bone marrow neutrophils were purified as previously described [27]. Femurs and tibias were collected from both hind limbs and freed of soft tissue. The bones were flushed through with ice cold KRG without Ca^2+^ and the collected cell suspension was filtered through a 40 µm nylon cell strainer and pelleted by centrifugation at 200 *g*. The pellet was resuspended in cold KRG without Ca^2+^ and laid on top of a three layer gradient, 2 ml each of 1.095, 1.085, and 1.070 g ml^−1^ Percoll solutions and centrifuged at 500 *g* for 30 min at 4°C without brake. The interface containing neutrophils between the first and the second layer (1.085/1.095 g ml^−1^) was collected, washed and then resuspended in KRG. The red blood cells were removed through hypotonic lysis. The purity of mature neutrophils (>80%) was determined by cytospin and May-Grunwald Giemsa staining; remaining cells seemed to be mainly different kinds of immature leucocytes in line with earlier results [28].

Neutrophil superoxide anion production was measured using the isoluminol-ECL system as described above using a six-channel Biolumat LB 9505 apparatus (Berthold technologies) with 4-ml disposable polypropene tubes and 0.9 ml reaction mixtures. After equilibration for 5 min at 37°C, 0.1 ml stimulus was added and light emission recoded continuously. The samples were stimulated with D-peptide (WKYMVm; 0.1 µM) [27], PMA (50 nM), or opsonized *S. aureus* (10 bacteria/cell).

Opsonized bacteria were obtained by washing *S. aureus* in PBS followed by dilution in HBSS, including 20% mouse serum and 0.1% gelatin (GHBSS), and incubated for 30 min in 37°C on a shaker. The bacteria were then washed twice in GHBSS and then diluted in GHBSS and counted in a Burker chamber.

#### Statistical analysis

Statistical analyses were performed using the SPSS software (version 15.0.1 for Windows). We used 2-samples Student's t-test; whenever Levene's test revealed unequal variance we used Welch's t-test, for comparisons of numeric data between two experimental groups. When adjustments for covariates were needed, e.g. when outcomes could not be pooled between experiments, we instead used ANCOVA. Logarithmic transformations were used when appropriate to ensure normal distribution of data. Mann-Whitney test was used when other tests were not appropriate. Log rank test were used for analysis of mortality. Fold changes were calculated as a ratio between mRNA expression and the geometrical mean of the control group (uninfected or infected LFD-fed mice) mRNA expression. Differences in *n* are due to different number of mice at the start of experiment, mortality or laboratory errors. All tests are two-sided. Data are arithmetic mean±SEM or geometrical mean when log data are used, unless otherwise stated, and *P *<0.05 was considered significant.

## Results

### Increased mortality in HFD-fed mice after *S. aureus* inoculation

**Figure 1:**
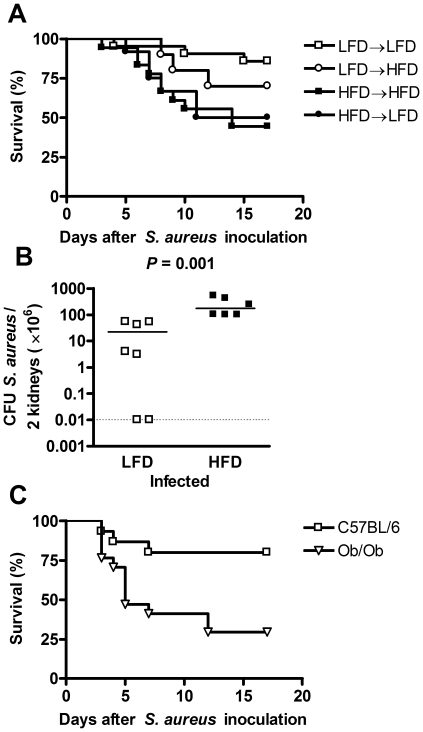
Survival and bacterial load after intravenous *S. aureus* inoculation. (A) Survival in mice fed: LFD before and after bacterial inoculation (LFD→LFD, *n* = 21), LFD before and HFD after bacterial inoculation (LFD→HFD, *n* = 10), HFD before and after bacterial inoculation (HFD→HFD, *n* = 18), HFD before and LFD after bacterial inoculation (HFD→LFD, *n* = 12). The HFD→HFD group had increased mortality, compared with LFD→LFD mice (*P* = 0.02). There was no difference between the LFD→HFD and LFD→LFD group (*P* = 0.9), or between the HFD→LFD group (*P* = 1). Log rank test. Data were collected from 2 different experiments. (B) Bacterial load in kidneys 5–7 days after bacterial inoculation in LFD- (*n* = 7) and HFD-fed (*n* = 6) mice. Mann-Whitney. Data are depicted as actual values or lowest/highest measurable value if not determinable. Detection limit is <0.01 CFU/2 kidneys (×10^6^), shown by the dotted line. Points represent individual mice; group median are denoted by a line throughout. (C) Survival in LFD-fed C57BL/6 (*n* = 15) and Ob/Ob (*n* = 18) mice after *S. aureus* inoculation. Ob/Ob mice had increased mortality compared with LFD-fed C57Bl/6 mice (*P* = 0.005). Log rank test.

After 8 weeks on HFD, C57BL/6 mice had increased body weight and fat mass compared with LFD-fed mice (Table S2). The *S. aureus*-induced mortality in mice fed HFD during the entire experiment (HFD→HFD) was increased when compared with mice on LFD during the whole period (LFD→LFD), an effect apparent at 5–7 days after bacterial inoculation (*P* = 0.02, Fig. 1A). This difference has been confirmed (data not shown). There was no increase in mortality when comparing mice that had been switched from LFD to HFD at the day of staphylococcal inoculation (LFD→HFD) with mice that had been fed LFD throughout (*P* = 0.9, Fig. 1A). Moreover, there was no significant difference in mortality between mice fed HFD before inoculation and switched to LFD at the day of inoculation (HFD→LFD), and mice that were on HFD throughout (*P* = 1, Fig. 1A).

To investigate if the increased mortality in HFD-fed mice was due to under- or over-reactivity of the immune system, we measured bacterial load in blood and kidneys on day 1 and in kidneys on days 5–7 after staphylococcal inoculation. There was no difference in bacterial load in either blood or kidneys 1 day after inoculation (Fig. S2). However, 5–7 days after inoculation there was a 10-fold increase in the number of staphylococcal CFU in the kidneys from HFD-fed mice, compared with LFD-fed mice (*P* = 0.001, Fig. 1B).

To investigate whether sepsis-induced mortality could be affected by obesity in the absence of HFD, genetically obese leptin deficient Ob/Ob mice were given LFD throughout the experiment. These obese mice showed increased mortality when infected with *S. aureus* compared with LFD-fed C57Bl/6 mice (*P* = 0.005, Fig. 1C).

### Increased cytokines in serum of infected HFD-fed mice

**Figure 2:**
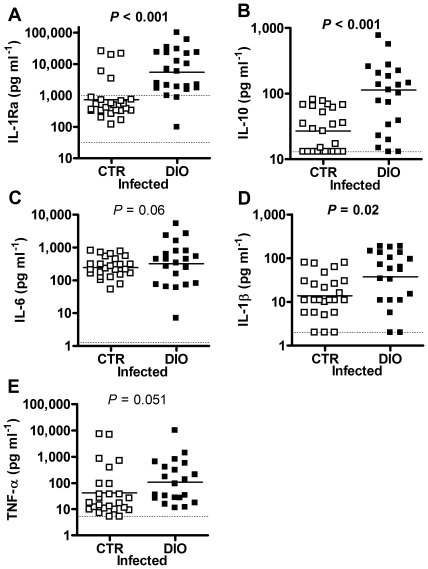
Cytokine levels in serum, 5–7 days after bacterial inoculation. (A) Serum levels of IL-1Ra in LFD- (*n* = 26) and HFD-fed (*n* = 23) mice. Detection limit is <31 pg/ml. Student's t-test on log data. (B) Serum levels of IL-10 in LFD- (*n* = 25) and HFD-fed (*n* = 21) mice. Detection limit is <13 pg/ml. Mann-Whitney. (C) Serum levels of IL-6 in LFD- (*n* = 25) and HFD-fed (*n* = 21) mice. Detection limit is <1.3 pg/ml. ANCOVA on log data with experiment as covariate. (D) Serum levels of IL-1β in LFD- (*n* = 25) and HFD-fed (*n* = 21) mice. Detection limit is <2 pg/ml. Student's t-test on log data. (E) Serum levels of TNF-α in LFD- (*n* = 25) and HFD-fed (*n* = 21) mice. Detection limit is <5.2 pg/ml. ANCOVA on log data with experiment as covariate. Data were collected from 3 different experiments. Data are depicted as actual values or lowest measurable value if not determinable; detection limit is denoted by the dotted line. In the scatter graphs, points represent individual mice; group means/medians are denoted by a line throughout.

We measured several serum cytokines in uninfected and infected mice. In uninfected mice the only difference was seen in the levels of the anti-inflammatory cytokine IL-1Ra, which were increased in HFD-fed mice (169 (range: 33–1111) and 1587 (range: 84–32245) pg ml^−1^, for LFD- vs. HFD-fed mice, *P*<0.001, ANCOVA on log data with experiment as covariate, data from 4 different experiments, *n* = 28+26). Infected HFD-fed mice had 4.9-fold increased levels of IL-1Ra (*P*<0.001) and also 4.7-fold increased levels of another anti-inflammatory cytokine, IL-10 (*P*<0.001, Fig. 2A and B). IL-6 has pro-inflammatory functions, but has also been found to have anti-inflammatory effects [29], [30]. This cytokine was increased in four HFD-fed mice, but overall not significantly (Fig. 2C). Among the pro-inflammatory cytokines measured, IL-1β was increased (*P* = 0.02) and there was a tendency for increased TNF-α in HFD-fed mice, compared with LFD-fed mice (Fig. 2D and E).

### Increased mRNA expression of cytokines in fat of HFD-fed mice

**Figure 3:**
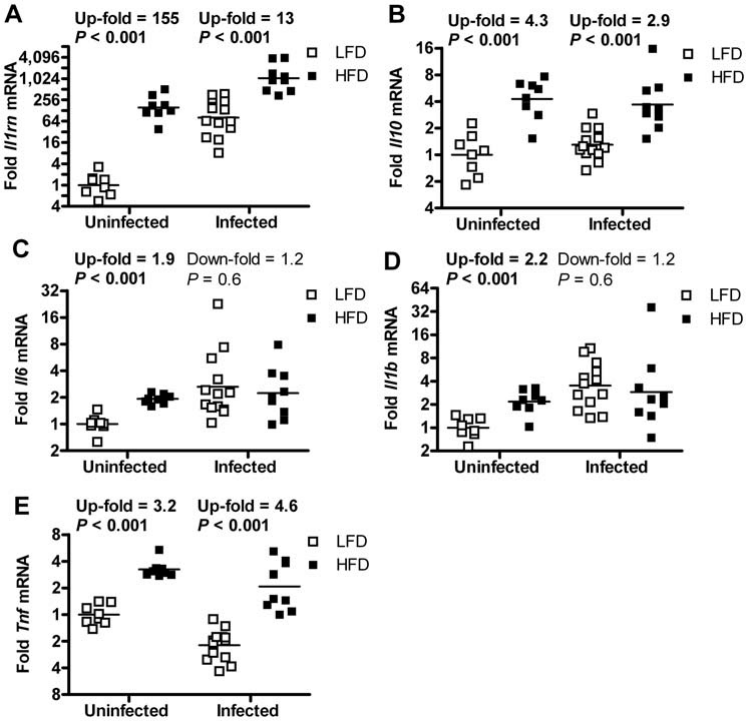
Cytokine mRNA expression in gonadal adipose tissue. (A–E) mRNA expression was measured in uninfected LFD- (*n* = 8) and HFD-fed (*n* = 8) mice, and in LFD- (*n* = 13) and HFD-fed (*n* = 9) mice 5–7 days after bacterial inoculation. mRNA from the following cytokine genes was measured: (A) *Il1rn*, (B) *Il10*, (C) *Il6*, (D) *Il1b*, and (E) *Tnf*,. Welch's test. In the scatter graphs, points represent individual mice (fold change as compared with uninfected LFD-fed mice); group geometrical means are denoted by a line throughout. Statistical comparisons are made between LFD- and HFD-fed mice in both infected and uninfected animals.

We investigated mRNA expression of several cytokines in fat, liver and spleen in uninfected and infected mice. All measured cytokines, both anti-and pro-inflammatory, were significantly up-regulated in fat from uninfected HFD-fed mice compared with uninfected LFD-fed mice (*P*<0.001, Fig. 3A–E). The largest increase was seen in IL-1Ra gene (*Il1rn*) expression, which was up regulated 155-fold. In liver we also found an increase in *Il1rn* (*P = *0.03, Fig. S3A) and a decrease of *Il6* and *Il1b* in uninfected HFD-fed mice (*P≤*0.001, Fig. S3C and D), while there was no difference in spleen of uninfected mice (Fig. S4A–E).

In infected mice, there were 13- and 2.9-fold increases of the anti-inflammatory cytokines *Il1rn* and *Il10*, respectively in fat from HFD-fed mice as compared with LFD-fed mice (*P*<0.001, Fig. 3A and B). The expression of the pro-inflammatory cytokine *Tnf* was increased 4.6-fold in HFD-fed mice (*P*<0.001), whereas the expression of *Il1b* and *Il6* did not differ between groups (Fig. 3C*–*E). In liver from infected HFD-fed mice there was a 2.6-fold decrease in *Il1b* expression (*P*<0.001, Fig. S3D). As in uninfected mice, there was no difference in expression in the spleen (Fig. S4A–E).

The relative increase in *Il1rn* mRNA (HFD versus LFD) was 155-fold in uninfected and only 13-fold in infected mice (Fig. 3A). However, the absolute difference between LFD and HFD was 154 units before infection and 974 units after infection. Based on this calculation, the difference in *Il1rn* expression between LFD and HFD would actually be larger rather than smaller after infection.

#### Increased proportion but decreased function of monocytes in HFD-fed mice

**Figure 4:**
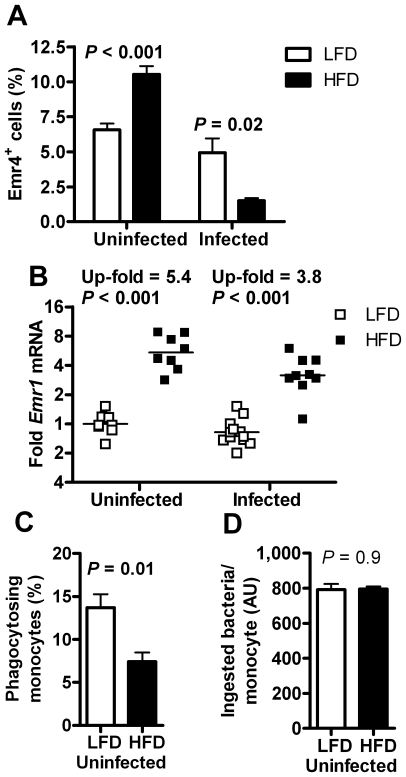
Monocyte proportion and function. (A) Percentage of monocytes (Emr4^+^ cells) in blood, as determined by FACS, in uninfected LFD- (*n* = 5) and HFD-fed (*n* = 5) mice (Student's t-test), and 5–7 days after bacterial inoculation in LFD- (*n* = 7) and HFD-fed (*n* = 7) mice (Welch's t-test). (B) *Emr1* mRNA expression in gonadal adipose tissue in uninfected LFD- (*n* = 8) and HFD-fed (*n* = 8) mice, and in LFD- (*n* = 12) and HFD-fed (*n* = 9) mice 5–7 days after bacterial inoculation. Welch's t-test. (C) Percentage of phagocytosing monocytes in uninfected LFD- (*n* = 5) and HFD-fed mice (*n* = 5). Welch's t-test. (D) Ingested bacteria per monocyte in uninfected LFD- (*n* = 5) and HFD-fed (*n* = 5) mice. Student's t-test. In the scatter graphs, points represent individual mice (fold change as compared with uninfected LFD-fed mice); group geometrical means are denoted by a line throughout. Statistical comparisons are made between LFD- and HFD-fed mice in both infected and uninfected animals.

FACS analysis of the monocyte/macrophage marker Emr4 (also called FIRE) showed a 60% increase (*P*<0.001) in the blood proportion of monocytes in uninfected HFD-fed mice compared with LFD-fed mice, but after bacterial inoculation there was instead a 70% decrease (*P* = 0.02, Fig. 4A). Similar results were obtained when measuring another marker of monocytes/macrophages Emr1 (also known as F4/80, data not shown). The mRNA expression of *Emr1* in fat was increased 6.3-fold (*P* = 0.003) in uninfected HFD-fed mice compared with LFD-fed mice, as expected [10], [11]. Also infected HFD-fed mice had increased *Emr1* expression in fat (*P*<0.001, Fig. 4B).

Uninfected HFD-fed mice had a lower proportion of phagocytosing monocytes than LFD-fed mice (*P* = 0.01, Fig. 4C). However, a measure of ingested number of bacteria per monocyte did not differ between groups (Fig. 4D). Phagocytic activity in infected mice could not be determined due to small samples and few monocytes.

The proportion of blood T- and B-cells measured by FACS in uninfected mice did not differ between groups (data not shown).

#### Decreased neutrophil proportion and function in HFD-fed mice

**Figure 5:**
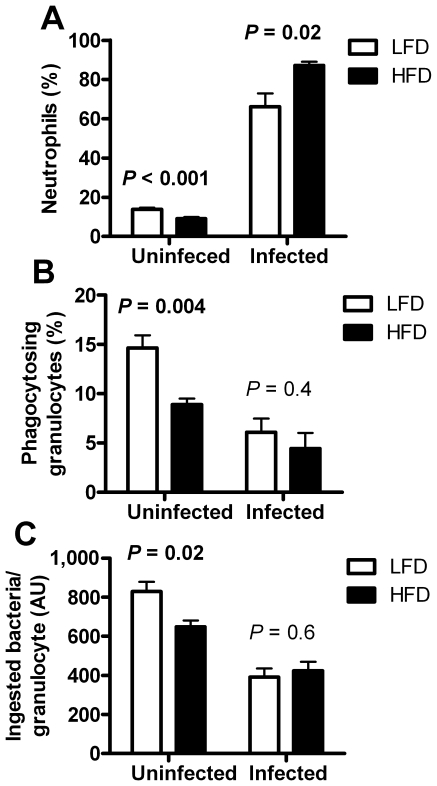
Granulocyte proportion and function. (A) Percentage of granulocytic neutrophils as determined by differential counts, in uninfected LFD- (*n* = 26) and HFD-fed (*n* = 29) mice (Student's t-test, data are collected from 2 different experiments), and 5–7 days after bacterial inoculation in LFD- (*n* = 7) and HFD-fed (*n* = 7) mice (Welch's t-test). (B) Percentage of phagocytosing granulocytes in uninfected LFD- (*n* = 5) and HFD-fed mice (*n* = 5) (Student's t-test), and 5–7 days after inoculation in LFD- (*n* = 14) and HFD-fed (*n* = 13) mice (Student's t-test on log data that were collected from 2 different experiments). (C) Ingested bacteria per granulocyte in uninfected LFD- (*n* = 5) and HFD-fed mice (*n* = 5) (Student's t-test), and 5–7 days after bacterial inoculation in LFD- (*n* = 14) and HFD-fed (*n* = 13) mice (Student's t-test on log data that were collected from 2 different experiments).

There was a decrease in the percentage (*P*<0.001) of neutrophilic granulocytes in the blood of uninfected HFD-fed mice, but 5–7 days after bacterial inoculation there was instead an increase (*P* = 0.02), compared with LFD-fed mice (Fig. 5A).

Granulocytes were tested for phagocytic activity both in uninfected mice and 5–7 days after bacterial inoculation. In the uninfected HFD-fed mice the proportion of granulocytes that were able to phagocytize staphylococci was decreased by 40%, compared with LFD-fed mice (*P* = 0.004). However, 5–7 days after bacterial inoculation there was no difference between groups (Fig. 5B). Furthermore, granulocytes from uninfected HFD-fed mice ingested fewer bacteria per granulocyte (*P* = 0.02, Fig. 5C).

#### Decreased phagocytic ROS production in HFD-fed mice

**Figure 6:**
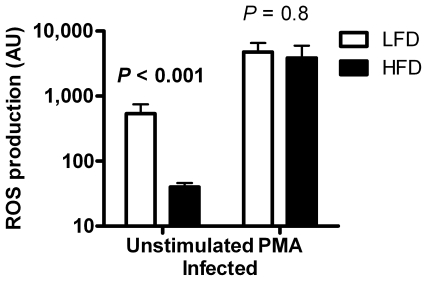
ROS production in lavage cells. ROS production 5–7 days after bacterial inoculation in LFD- (*n* = 7) and HFD-fed (*n* = 6) mice. Peritoneal lavage cells were left unstimulated or stimulated with or PMA. ANCOVA on log data with experimental day as covariate.

Neutrophilic granulocytes and macrophages are both capable of ROS production that contributes to microbial killing. We measured the capacity of peritoneal lavage cells, containing neutrophils, macrophages and lymphocytes, to secrete ROS 5–7 days after *S. aureus* inoculation. Unstimulated lavage cells from HFD-fed mice produced only 7% of the ROS produced in lavage cells from LFD-fed mice (P<0.001, Fig. 6). In contrast, the maximal capacity of ROS production, as measured after PMA stimulation, did not differ between groups (Fig. 6). The cell composition of lavage samples did not differ significantly between the different diets (not shown).

To find out if the ability to produce ROS was altered already before entering the blood circulation we isolated granulocytes from bone marrow. The granulocytes were then stimulated with D-peptide, live opsonized *S. aureus*, or PMA. No differences in ROS production were detected between cells from HFD-fed and LFD-fed mice (data not shown).

#### Decreased spleen weight and changed spleen leukocyte population in HFD-fed mice

**Figure 7:**
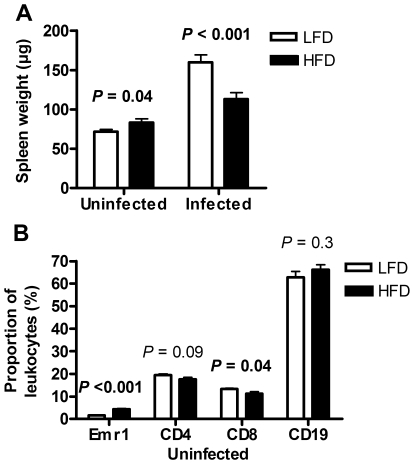
Spleen weight and leukocyte fractions. (A) Spleen weight in uninfected LFD- (*n* = 13) and HFD-fed (*n* = 13) mice, and 5–7 days after inoculation in LFD- (*n* = 16) and HFD-fed (*n* = 16) mice. Student's t-test on log data. Data were collected from 2 different experiments. (B) Spleenic leukocyte fractions in uninfected LFD- (*n* = 7) and HFD-fed (*n* = 8) mice. Emr1 (Welch's t-test), CD4 (Student's t-test), CD8 (Student's t-test), and CD19 (Student's t-test) were used as markers for monocytes/macrophages, T-helper, T cytotoxic, and B cells respectively.

The spleen weight in uninfected mice was increased in HFD-fed mice compared with LFD-fed mice (*P* = 0.04, Fig. 7A). After body weight adjustment, this difference was however not significant (*P* = 0.07). The spleens from HFD-fed mice contained more white blood cells than those from LFD-fed mice (34.5±3.1 vs. 22.4±2.3 (×10^9^ l^−1^), *P* = 0.009, Student's t-test, *n* = 7+8). However, after adjustment for spleen weight, this difference lost significance (*P* = 0.1). Furthermore, the spleen weight was 40% lower in HFD-fed mice, compared with LFD-fed mice, when weighed 5–7 days after bacterial inoculation (*P*<0.001, Fig. 7A). The decreased spleen weight was also highly significant after body weight adjustment (*P* = 0.004).

The leukocyte population in the spleen was examined by FACS. The monocyte proportion (Emr1^+^ cells) was 2.8-fold higher in uninfected HFD-fed mice than in LFD-fed mice (*P*<0.001, Fig. 7B). The cytotoxic T (CD8^+^)-cell proportion was slightly lower in HFD-fed mice than in LFD-fed mice (*P* = 0.04), while there was no difference in B (CD19^+^)-cell or T (CD4^+^)-helper cell proportion between groups (Fig. 7B).

## Discussion

HFD-fed mice had increased delayed mortality in *S. aureus*-induced sepsis from about one week after bacterial inoculation. Acute administration of HFD at the time of inoculation did not lead to increased mortality. This indicates that the increased mortality in HFD-fed mice is due to chronic effects of the diet, for example obesity, rather than short-term effects of the HFD. The finding that genetically-induced obese Ob/Ob mice given LFD also display increased mortality supports this notion. The increased mortality appears to be due to an under reactivity of the innate immune system, as an increased bacterial load was found in the kidneys of HFD-fed mice. The reason for this disturbed innate immune function is unknown, but we observed several possible mechanisms. The anti-inflammatory cytokine IL-1Ra was increased in serum and fat in both uninfected and infected HFD-fed mice compared with LFD-fed mice. The other measured anti-inflammatory cytokine, IL-10, was increased in serum in infected HFD-fed mice, and in fat in uninfected and infected HFD-fed mice. However, the serum levels of the pro-inflammatory cytokine IL-1β were also increased, while the levels of the TNF-α and IL-6 tended to be increased in HFD-fed mice. In summary, both pro-inflammatory and anti-inflammatory cytokines were enhanced in mice given HFD. In addition, both the proportion and function of granulocytes were decreased in uninfected HFD-fed mice, and the ROS production by phagocytosing cells was greatly decreased in infected HFD-fed mice. Therefore, several immune functions were disturbed in chronically HFD-fed mice.

The finding that the HFD-fed mice had some signs of decreased immune response during infection was surprising, as data in the literature clearly show that uninfected HFD-fed mice have a chronic inflammation, i.e. stimulated immune functions [9]–[12], [31], [32]. Although the immune system of obese individuals is thus activated already at the time of infection, our results show that this chronic activation does not facilitate immune system activation and pathogen clearance during infection. If anything, chronic inflammation seems associated with impaired effectiveness of the immune system. In line with our findings, the autoimmune diseases rheumatoid arthritis and systemic lupus erythematosus are associated with increased risk of acquiring infections, an event seemingly independent of immunosuppressive treatments [33], [34].

Obesity has been shown to up-regulate production of both pro- and anti-inflammatory cytokines, from adipocytes and/or fat infiltrating macrophages [9]–[12], [35]–[37], as consistent with the present findings in uninfected HFD-fed mice. The anti-inflammatory cytokines IL-1Ra and IL-10 were both markedly up-regulated in fat and serum in HFD-fed mice on day 5–7 after bacterial challenge. At this time point HFD-fed mice have increased bacterial load and their mortality starts to increase. IL-1β was the only measured pro-inflammatory cytokine that then was higher in serum of HFD-fed mice, although there was a tendency for increased TNF-α and IL-6. The relatively large up-regulation of anti- versus pro-inflammatory cytokines in serum may be important for the decreased survival of HFD-fed mice in this study. The mortality in clinical sepsis often occurs days to weeks after the first clinical event and is associated with a shift toward an immunosuppressive state [3], [4]. In fact, it has been shown that IL-1Ra treatment before inoculation enhances mortality in experimental sepsis [38] and IL-10 neutralization has also been shown to enhance survival to sepsis [39].

In the present study we confirm earlier findings that uninfected HFD-fed mice accumulate macrophages in fat [10], [11]. They also have higher monocyte/macrophage proportion in blood and spleen. When infected, HFD-fed mice still have an accumulation of macrophages in their fat. However, infected HFD-fed mice had a lower monocyte proportion in blood, and the proportion of phagocytosing monocytes was decreased in uninfected HFD-fed mice. At present, it is unclear to what extent decreased macrophage function contributes to decreased immune response to *S. aureus* in HFD-fed mice.

We observed several signs of decreased granulocyte/neutrophil efficacy in HFD-fed mice, including decreased proportion in blood of neutrophils, decreased proportion of phagocytosing granulocytes, and fewer ingested bacteria per granulocyte in uninfected HFD-fed mice. There was also a marked decrease in ROS production by phagocytic cells in infected HFD-fed mice. Neutrophilic dysfunction clearly contributes to increased bacterial growth and increased mortality [40]–[42], reasonably also in HFD-fed mice. Indeed, neutrophils, the major subpopulation of granulocytes, are often first to migrate into tissues in response to invading pathogens and to attack bacteria in the blood circulation [43], [44]. Neutrophilic depletion in mice leads to severely increased mortality in *S. aureus*-induced sepsis [22]. Furthermore, most patients with congenital neutropenia, also known as Kostmann syndrome, die from bacterial infections in early childhood, unless properly treated [45]. These findings emphasize the importance of neutrophils in the early clearance of bacteria. Interestingly, there are indications that neutrophil function and ROS production is of importance not only to overcome an infection, but also for the subsequent resolution of the inflammation [43], [46], [47]. The role of neutrophil function for HFD-associated inflammation remains to be further investigated.

In line with our study, Leeman and coworkers recently reported that HFD-induced obesity aggravates a local infection, the periodontitis caused by *Porphyromonas gingivalis* [48]. These results are of interest as it is well established that periodontitis is associated with obesity [49]. Taken together with our findings it appears that the immune response in general is decreased by HFD both with regard to low-grade chronic local infection and acute life-threatening generalized infection.

A limitation with the use of HFD-fed mice is that it is difficult to determine whether it is the diet itself, or secondary causes thereof, such as obesity or blood fat disturbances that influence the immune system. For example, Kopf et al found that several immune related parameters change in *Apoe^−/−^* mice given high fat high cholesterol diet, as a model of dyslipidemia [50]. In this study we fed C57BL/6 mice a HFD with a 30-time lower cholesterol content, that do not cause major dyslipidemia in this strain [51]. Thus blood fat disturbances are an unlikely mediator of immune suppression in our HFD-fed mice. Differences in diet may also affect immune functions indirectly via effects on bacterial load instead of *vice versa*. Furthermore, there is always a possibility of species differences between mice and humans. For instance, the proportion of neutrophils was decreased by HFD in uninfected mice in this study, while neutrophils in blood seem to be higher in obese humans [52].

In the present study we found increased mortality not only in obese HFD-fed mice, but also in genetically obese Ob/Ob mice that had not been given HFD. Complete lack of leptin activity is associated with immune deficiencies in both mice and humans, e.g. suppression of T-cells [53], [54]. Therefore, it is possible that the reason for increased mortality differ between Ob/Ob and HFD-fed mice. This is supported by the fact that IL-1Ra and IL-10 response after LPS exposure is decreased in Ob/Ob mice [55], although we observed higher levels of IL-1Ra and IL-10 in HFD-fed obese mice in the present study. It should be noted that complete lack of leptin activity is a very rare cause of severe obesity in humans, while HFD-fed mice is generally accepted as a good model for the common clinical obesity due to the combined effect of high caloric density diet and multigenic predisposition [20], [21].

Like obesity, sepsis and especially that caused by *S. aureus* is increasing worldwide [1], [2] and it is therefore urgent to understand basic mechanisms and find better treatments for this condition. The present study shows for the first time that HFD-fed mice, despite their well known low-grade inflammation, have increased mortality and bacterial proliferation in connection to sepsis. These effects occurred several days after bacterial challenge, i.e. at a time when mortality often occurs in clinical sepsis, not seldom because of hypoinflammation [3], [4]. The HFD-fed mice had disturbed innate immune functions, as indicated by increased levels of immune suppressing and immune stimulating cytokines and decreased granulocyte function. Based on successful animal studies, sepsis treatment in clinical trials has often sought to decrease an early overactive immune response, but these drugs have been largely ineffective [3]–[5]. Experimental studies have usually been performed on lean young animals, while patients with sepsis on average are 55–60 years old and often have several symptoms of the metabolic syndrome [1]. We suggest that experimental sepsis studies should be conducted in models that are metabolically more similar to the clinical situation.

## Supporting Information

Table S1 Primer and assays used for real-time RT PCR. F: forward primer, R: reverse primer, *Tataa Biocenter, Gothenburg, Sweden
(0.03 MB DOC)

Table S2 Body weight, lean mass, and fat mass in 14 weeks old male C57BL/6 mice after 8 weeks of LFD or HFD.
(0.03 MB DOC)

Figure S1 Flow cytometry plot gated with fluorochrome minus one (FMO). Cells were obtained for a 4 color flow cytometry analysis and stained as described in material and methods. By removing one flourochrome from each staining, a negative control (FMO) was obtained. FMO for (A) peridinin-chlorophyll-protein (PerCP; CD8), (B) allophycocyanin (APC; CD4), (C) FITC (Emr1) and (D) pacific blue (CD19) are shown as dot plots. Gating were performed on mononuclear cells and then analyzed for the background levels in the empty channel of each fluorochrome.
(1.71 MB TIF)

Figure S2 Bacterial load after intravenous *S. aureus* inoculation. (A) Bacterial load in blood 1 day after bacterial inoculation in lean control (n = 8) and DIO (n = 6) mice. Mann-Whitney. (B) Bacterial load in kidneys 1 day after bacterial inoculation in lean control (n = 8) and DIO (n = 6) mice. Welch's test.
(1.59 MB TIF)

Figure S3 **Cytokine mRNA expression in liver.** (A–E) mRNA expression was measured in uninfected LFD- (n = 8) and HFD-fed (n = 8) mice, and in LFD (n = 14) and HFD-fed (n = 11) mice 5–7 days after bacterial inoculation. mRNA from the following cytokine genes were measured: (A) *Il1rn*, (B) *Il10*, (C) *Il6*, (D) *Il1b*, and (E) *Tnf*. Welch's test. In the scatter graphs, points represent individual mice (fold change as compared with uninfected LFD-fed mice); group geometrical means are denoted by a line throughout. Statistical comparisons are made between LFD- and HFD-fed mice in both infected and uninfected animals.
(5.34 MB TIF)

Figure S4 **Cytokine mRNA expression in spleen.** (A–E) mRNA expression was measured in uninfected LFD- (n = 8) and HFD-fed (n = 8) mice, and in LFD- (n = 12) and HFD-fed (n = 9) mice 5–7 days after bacterial inoculation. mRNA from the following cytokine genes were measured: (A) *Il1rn*, (B) *Il10*, (C) *Il6*, (D) *Il1b*, and (E) *Tnf*. Welch's test. In the scatter graphs, points represent individual mice (fold change as compared with uninfected LFD-fed mice); group geometrical means are denoted by a line throughout. Statistical comparisons are made between LFD- and HFD-fed mice in both infected and uninfected animals.
(5.50 MB TIF)
