# Supplementary material for: Mice Chronically Fed High-Fat Diet Have Increased Mortality and Disturbed Immune Response in Sepsis
Source: PLoS One. 2009 Oct 28;4(10):e7605. doi: 10.1371/journal.pone.0007605 (PMC2765728; doi:10.1371/journal.pone.0007605)
Supplement: Table S1 — Primer and assays used for real-time RT PCR. F: forward primer, R: reverse primer, *Tataa Biocenter, Gothenburg, Sweden (0.03 MB DOC) [file pone.0007605.s001.doc]

| **Gene** | **Primer sequence (5’-3’) or assay number** | **Reference sequence** |
| --- | --- | --- |
| ***Eef2*** | Assay from Qiagen, QT01782816 | NM_007907.1 |
| ***Emr1*** | TaqMan® Gene Expression Assays from Applied Biosystems, ID Mm01233109_m1 | NM_010130.4 |
| ***Il1b*** | Assay from Tataa*, ID G112 | NM_008361 |
| ***Il1rn*** | Assay from Tataa, ID G113 | NM_031167 |
| ***Il6*** | TaqMan® Gene Expression Assays from Applied Biosystems, ID Mm00446190_m1 | NM_031168.1 |
| ***Tnf*** | F: CATCTTCTCAAAATTCGAGTGACAA | NM_013693.2 |
|  | R: TGGGAGTAGACAAGGTACACCCC |  |
