# Supplementary material for: Mice Chronically Fed High-Fat Diet Have Increased Mortality and Disturbed Immune Response in Sepsis
Source: PLoS One. 2009 Oct 28;4(10):e7605. doi: 10.1371/journal.pone.0007605 (PMC2765728; doi:10.1371/journal.pone.0007605)
Supplement: Table S2 — Body weight, lean mass, and fat mass in 14 weeks old male C57BL/6 mice after 8 weeks of LFD or HFD. (0.03 MB DOC) [file pone.0007605.s002.doc]

|  | **LFD (*n* = 22)** | **HFD (*n* = 21)** | ***P*** |
| --- | --- | --- | --- |
| **Body weight (g)** | 28.8 ± 0.5 | 39.3 ± 1.1 | <0.001 |
| **Lean mass (g)** | 20.7 ± 0.4 | 20.0 ± 2.4 | 0.2 |
| **Fat mass (g)** | 5.5 ±0.2 | 17.0 ± 0.6 | <0.001 |
