# Supplementary figures and images for: Mice Chronically Fed High-Fat Diet Have Increased Mortality and Disturbed Immune Response in Sepsis
Source: PLoS One. 2009 Oct 28;4(10):e7605. doi: 10.1371/journal.pone.0007605 (PMC2765728; doi:10.1371/journal.pone.0007605)

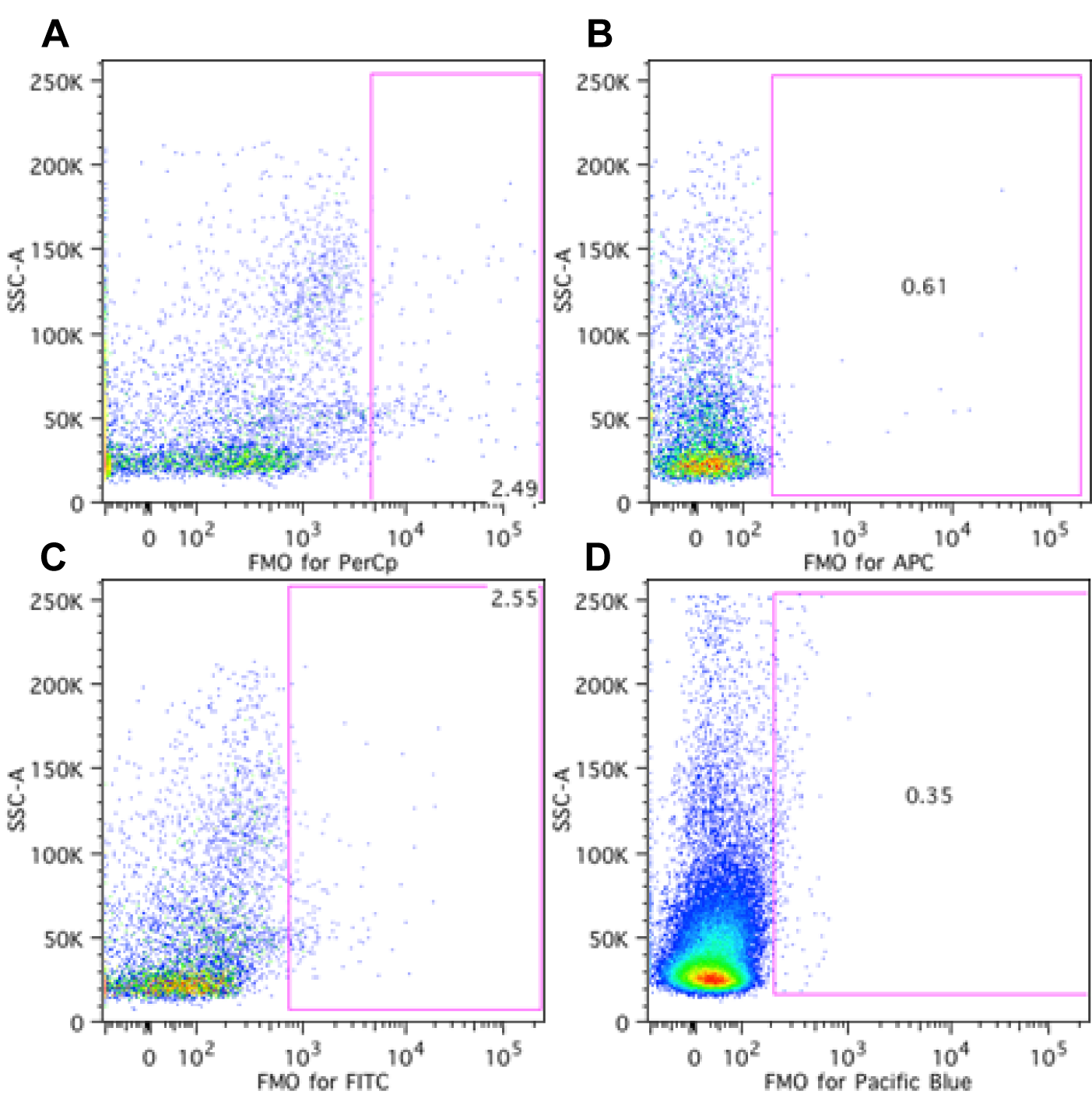

Supplement: Figure S1 — Flow cytometry plot gated with fluorochrome minus one (FMO). Cells were obtained for a 4 color flow cytometry analysis and stained as described in material and methods. By removing one flourochrome from each staining, a negative control (FMO) was obtained. FMO for (A) peridinin-chlorophyll-protein (PerCP; CD8), (B) allophycocyanin (APC; CD4), (C) FITC (Emr1) and (D) pacific blue (CD19) are shown as dot plots. Gating were performed on mononuclear cells and then analyzed for the background levels in the empty channel of each fluorochrome. (1.71 MB TIF) [file pone.0007605.s003.tif]
